# Kidney Transplantation from Donors with Resolved HBV Infection: Minimal but Potential Transmission Risk and the Need for Improved Recipient Vaccination

**DOI:** 10.3390/jcm15103846

**Published:** 2026-05-16

**Authors:** Taeko Sasaki, Shigeyoshi Yamanaga, Yuji Hidaka, Yoei Miyabe, Chiaki Kawabata, Mariko Toyoda, Yu Watanabe, Yasuhiro Yamamoto, Akito Inadome, Hiroshi Yokomizo

**Affiliations:** 1 Department of Surgery, Japanese Red Cross Kumamoto Hospital, 2-1-1 Nagamineminami, Higashi-ku, Kumamoto 861-8520, Japan; taetae219@gmail.com (T.S.);; 2 Department of Nephrology, Japanese Red Cross Kumamoto Hospital, 2-1-1 Nagamineminami, Higashi-ku, Kumamoto 861-8520, Japan; 3 Department of Urology, Japanese Red Cross Kumamoto Hospital, 2-1-1 Nagamineminami, Higashi-ku, Kumamoto 861-8520, Japan

**Keywords:** kidney transplantation, anti-HBc-positive donors, HBV transmission risk, HBV vaccination

## Abstract

**Background/Objectives**: Hepatitis B virus (HBV) transmission risk from donors with resolved HBV infection remains a concern in kidney transplantation, because covalently closed circular deoxyribonucleic acid (DNA) may persist despite serological recovery. In this study, we evaluated the transmission risk from anti-hepatitis B core antibody (anti-HBc)-positive, hepatitis B surface antigen (HBsAg)-negative, and HBV-DNA-negative living donors to anti-HBc-negative recipients. **Methods**: We retrospectively reviewed living-donor kidney transplantations performed at our institution (June 2011–December 2024). Among 277 transplantations, 26 met the inclusion criteria. All recipients had received HBV vaccination prior to transplantation. Serological markers, including HBsAg, anti-HBc, anti-hepatitis B surface antibody (anti-HBs), and HBV-DNA, were monitored before and after transplantation. **Results**: Five recipients (19.2%) achieved anti-HBs seroconversion after vaccination pre-transplantation, but all lost protective antibody levels before or shortly after transplantation. Five recipients (19.2%) showed transient post-transplantation anti-HBs positivity; three cases were considered vaccine-related, and two were possibly transplant-related. None of the patients developed liver enzyme elevation, clinical hepatitis, HBsAg positivity, or anti-HBc seroconversion. Among the four recipients tested for HBV-DNA, all results remained undetectable throughout follow-up. None of the recipients maintained protective anti-HBs levels pre- or post-transplantation, despite universal vaccination. Two recipients (7.7%) developed delayed anti-HBs positivity without anti-HBc seroconversion, HBsAg positivity, or detectable HBV-DNA, suggesting possible subclinical HBV antigen exposure from the allograft. **Conclusions**: Kidney transplantation from donors with resolved HBV infection was not associated with clinically evident HBV infection, although transient serological changes were observed. Optimized vaccination strategies and structured post-transplant monitoring may enhance the safety of transplantations involving such donors.

## 1. Introduction

Kidney transplantation is the definitive treatment for end-stage renal disease and is known to provide superior patient survival and quality of life compared to renal replacement therapies, such as hemodialysis (HD) and peritoneal dialysis (PD) [1]. Because lifelong immunosuppressive therapy is required to prevent graft rejection, transplant recipients are at risk of infection. Therefore, protection against vaccine-preventable diseases should be initiated before transplantation, given the potential for donor-to-recipient transmission [2].

An estimated 1.0–1.3 million individuals in Japan have serological evidence of past hepatitis B virus (HBV) infection [3]. HBV is known to persist as covalently closed circular deoxyribonucleic acid (cccDNA) in hepatocytes, even after serum hepatitis B surface antigen (HBsAg) becomes negative following acute infection [4]. In liver transplantation using hepatitis B core antibody (anti-HBc)-positive donors, the incidence of de novo HBV infection among HBV-naïve recipients has been reported to be as high as 47.8% [5], and well-established management protocols, including the use of lamivudine, are in place because of the high risk of HBV transmission [6]. Although rare in kidney transplantation, the rate of transmission is reportedly 0–26.6% [7,8,9,10,11,12,13,14,15,16,17,18,19,20,21]. Traditionally, HBV has been thought to persist in the lymphoid tissue of anti-HBc-positive, hepatitis B virus deoxyribonucleic acid (HBV-DNA)-negative allografts [22]. Recently, cccDNA was detected in 3.1% (1/32) of renal graft tissues from donors with resolved HBV infection, and 4.4% (2/45) of recipients were suspected of being transmitted through the allograft [21]. Based on these advances in the understanding of HBV transmission from anti-HBc-positive, HBV-DNA-negative donors, a national guideline for preventing HBV transmission from anti-HBc-positive donors in non-liver solid organ transplantation was proposed in 2023 in Japan [23]. However, studies on this topic are limited.

This study aimed to evaluate the incidence of changes in HBV serological markers and clinical hepatitis in anti-HBc-negative recipients of living-donor kidney transplants from donors with resolved HBV infection at our institution.

## 2. Materials and Methods

We retrospectively reviewed living-donor kidney transplantations performed at our institution between June 2011 and December 2024, from anti-HBc-positive donors to anti-HBc-negative recipients. Data were collected from electronic medical records. Donor variables included age, sex, HBsAg status, and HBV-DNA. Recipient variables included age, sex, primary kidney disease, pre-transplant dialysis modality and duration, ABO compatibility, use and dose of rituximab, HBV vaccination history, changes in anti-HBs titers before and after transplantation, and post-transplant changes in HBsAg, anti-HBc, and HBV-DNA. The follow-up duration, liver enzyme elevation, graft loss, and mortality were also recorded. Liver enzyme elevation was defined as an alanine aminotransferase (ALT) level ≥100 IU/L based on previous reports [21].

### 2.1. Exclusion Criteria

Recipients who were anti-HBs-positive before transplantation without a documented HBV vaccination history were excluded, because anti-HBs positivity in these cases could not be confidently attributed to vaccination versus prior HBV infection. Patients who were lost to follow-up due to relocation were also excluded.

### 2.2. Immunosuppressive and Desensitization Protocols

The standard immunosuppressive regimen consists of tacrolimus, mycophenolate mofetil (MMF), and methylprednisolone. Desensitization therapy comprised rituximab, plasmapheresis, and MMF.

### 2.3. Assessment of Serological Markers

#### 2.3.1. anti-HBc

From 2011 to May 2015, anti-HBc levels were measured using the Lumipulse Forte system (Fujirebio, Tokyo, Japan) with a chemiluminescent enzyme immunoassay (CLEIA). Results with <50% inhibition (INH) were considered negative.

From June 2015 to 11 March 2022, the Lumipulse Presto II system (Fujirebio, Tokyo, Japan) was used with CLEIA, and values < 1.0 cutoff index (COI) were defined as negative.

From 12 March 2022 onward, anti-HBc was measured using the Alinity system (Abbott Japan, Tokyo, Japan) with a chemiluminescent immunoassay (CLIA); values < 1.0 signal-to-cutoff ratio (S/CO) were considered negative.

#### 2.3.2. anti-HBs

From 2011 to 2013, anti-HB levels were measured using an AxSYM system (Abbott Japan, Tokyo, Japan) with a microparticle enzyme immunoassay (MEIA). Values < 5 mIU/mL were considered negative.

From 2014 to 11 October 2018, the ARCHITECT system (Abbott Japan, Tokyo, Japan) was used with CLIA, and values < 10 mIU/mL were defined as negative. 

From 12 October 2018 to 11 March 2022, anti-HBs were measured using the Lumipulse Presto II system (Fujirebio, Tokyo, Japan) with CLEIA; values < 10 mIU/mL were considered negative. 

From 12 March 2022 onward, anti-HBs were measured using the Abbott Japan assay based on CLEIA, with values < 10 mIU/mL defined as negative.

#### 2.3.3. HBsAg

HBsAg levels were measured during the same period using the same analyzers as those used for anti-HBs. However, owing to differences in assay characteristics, the cutoff values and measurement units for defining negativity differed from those of anti-HBs.

#### 2.3.4. HBV-DNA

Until July 2024, HBV-DNA testing was outsourced, and the specific assay platforms could not be identified; samples were considered negative if HBV-DNA was not detected by polymerase chain reaction (PCR). 

From August 2024, HBV-DNA was measured using a Cobas 5800 system (Roche Diagnostics, Rotkreuz, Switzerland), and the results were considered negative when HBV-DNA was undetectable by PCR.

## 3. Results

### 3.1. Screening of Study Patients

**Figure 1:**
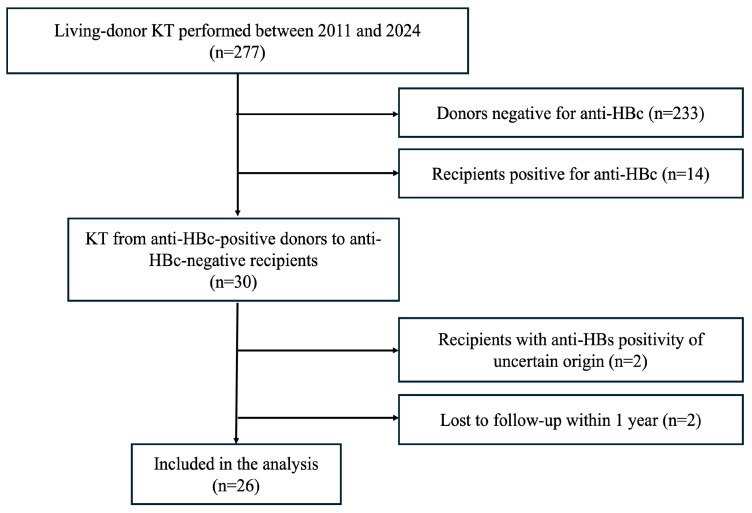
Flow diagram of study participant selection. anti-HBc—hepatitis B core antibody; anti-HBs—hepatitis B surface antibody; KT—kidney transplantations.

Among the 277 living-donor kidney transplantations performed during the study period, 30 were from anti-HBc-positive donors to anti-HBc-negative recipients. Two recipients with uncertain vaccination status (hepatitis B surface antibody (anti-HBs)-positive before transplantation) and two recipients lost to follow-up within one year were excluded, leaving 26 cases for analysis (Figure 1).

### 3.2. Baseline Characteristics

The median donor age was 67.0 years; 11 donors were male, and 15 were female. All donors tested negative for HBsAg and HBV-DNA. The median recipient age was 47.5 years; 20 recipients were male, and 6 were female. Primary kidney diseases included diabetic nephropathy (n = 9), glomerulonephritis (n = 6), immunoglobulin A (IgA) nephropathy (n = 5), combined glomerulonephritis and diabetic nephropathy (n = 1), IgA nephropathy or diabetic nephropathy (n = 1), renal tuberculosis (n = 1), mitochondrial nephropathy (n = 1), focal segmental glomerulosclerosis (n = 1), and unknown etiology (n = 1).

Pre-transplant renal replacement therapy included HD in 10 patients, PD in 2, combined or transitioned HD/PD in 4, and pre-emptive kidney transplantation (PEKT) in 10. Among the 16 dialyzed recipients (excluding PEKT recipients), the median dialysis duration was 26 months. ABO-compatibility was present in 14 cases; all 12 ABO-incompatible cases received rituximab (400 mg in 4 cases and 200 mg in 8 cases) (Table 1).

### 3.3. Vaccination and Seroconversion

All 26 recipients received HBV vaccination before transplantation; however, only five achieved seroconversion pre-transplant, and all subsequently lost seropositivity over time. The vaccination consisted of three doses in 3 cases, two doses in 22 cases, and one dose in 1 case because of allergic reactions (Table 2). Post-transplantation, anti-HBs became transiently positive in five recipients (three men and two women; age range, 23–51 years). In three of these recipients, anti-HB positivity occurred within 3 months of the last pre-transplant vaccine dose. In the remaining two recipients (7.7%), anti-HBs positivity was first detected 2–3 years after the last vaccination. No recipient tested positive for HBsAg or anti-HBc after transplantation. Among the four recipients in whom HBV-DNA was tested, results remained undetectable (Table 3).

### 3.4. Follow-Up and Complication

All 26 recipients underwent post-transplant testing for HBsAg, anti-HBs, and anti-HBc at least once, although the testing frequency and intervals varied across patients and were not standardized. HBV-DNA was measured in only 4 of 26 recipients (15.4%). The remaining 22 recipients did not undergo HBV-DNA testing during the follow-up period. Five recipients experienced liver enzyme elevation, one experienced graft loss, and none died during follow-up (Table 3).

## 4. Discussion

In this study of 26 living-donor kidney transplants from donors with resolved HBV infection, none of the recipients developed clinically evident HBV infection. However, five recipients (19.2%) demonstrated post-transplant anti-HBs seroconversion. Among these, three cases occurred several months after vaccination, suggesting a vaccine-related increase. The remaining two cases (2/26; 7.7%) developed anti-HBs positivity years after the last vaccine dose, a timing pattern that is more consistent with allograft-related antigen exposure than with a delayed vaccine response. Of the two recipients, one ultimately experienced graft loss due to chronic rejection without liver enzyme elevation, indicating that HBV transmission was unlikely, whereas the other recipient showed no liver dysfunction and maintained good graft function.

A nationwide survey by the Japan Society for Transplantation (JST) reported suspected HBV transmission in 1.5% of 339 transplants using anti-HBc-positive, HBV-DNA-negative non-liver donors [24], suggesting that transmission, although rare, remains possible. However, the detailed clinical background of these cases (vaccination history, degree of immunosuppression, and anti-HB trends) has not been reported.

A MEDLINE search using the terms (“kidney*” OR “renal*”) AND (“transplant*”) AND (“donor*”) AND (“anti-HBc*” OR “resolved hepatitis B*”), limited to publications from 1995 to 2026, identified 15 studies. Reported transmission rates ranged from 0% to 26.6%, liver enzyme elevation occurred in 0–25% of cases, and severe or fatal hepatitis was rare [7,8,9,10,11,12,13,14,15,16,17,18,19,20,21] (Table 4). These two cases of hepatic failure reported by Jeon JM et al. were possibly affected by the recipient’s anti-HBc-positive status [18].

According to previous studies, the utilization of kidneys from anti-HBc-positive donors has become a vital strategy to expand the organ pool. While these donors are HBsAg-negative and HBV-DNA-negative, the risk of HBV transmission still exists due to the potential presence of occult HBV infection within the donor kidney. Early studies by Satterthwaite et al. [7] and Madayag et al. [8] established that while the risk of transmission exists, it is clinically manageable and does not preclude the use of such grafts.

A primary concern in using anti-HBc-positive donors is whether it compromises long-term transplant success. According to the large-scale database analyses by Fong et al. [10], have shown that donor anti-HBc status does not independently impact graft or patient survival. A study by Wang et al. [20] confirmed that graft loss and mortality rates among recipients of anti-HBc-positive kidneys are comparable to those receiving anti-HBc-negative kidneys. In several cohorts, graft loss rates remained within the expected range for standard transplants, supporting the safety of this donor pool [7,8,9,13,18,19,20,21].

Although comparisons across studies are limited owing to differing donor and recipient serological profiles, recipient immune status is a critical determinant of post-transplant HBV outcomes. Recipients with anti-HBs, whether through prior infection or vaccination, demonstrate a significantly lower risk of hepatic failure. Jeon et al. reported that the presence of anti-HBs confers a strong protective effect, with hepatic failure rates as low as 1.1–1.4% compared to 5.6% in anti-HBs-negative recipients [18]. Naïve recipients (HBsAg-negative, anti-HBc-negative, and anti-HBs-negative) remain at the highest risk. Recently, Yamada et al. have highlighted that even with modern protocols, a risk of seroconversion or infection (approximately 4.4%) persists, necessitating rigorous monitoring in this subgroup [21]. The KDIGO guidelines consider anti-HBs ≥ 10 mIU/mL as protective [25]; however, antibody levels may decline rapidly after transplantation due to immunosuppression. Some countries recommend titers ≥100 mIU/mL, and Chancharoenthana et al. reported zero transmission or hepatitis in recipients meeting this threshold even when receiving grafts from HBsAg-positive donors [26]. Therefore, appropriate antibody management is central to ensuring post-transplantation safety.

In Japan, the standard vaccination protocol involves a three-dose recombinant HBV vaccination series (0, 1, and 6 months) aimed at achieving an anti-HBs titer of ≥10 mIU/mL prior to transplantation [23]. In our cohort, the pre-transplant seroconversion rate was low (19.2%) despite universal vaccination. This may reflect both the limited number of vaccine doses administered (only 3 of 26 recipients received the standard three-dose series) and impaired immune responsiveness associated with advanced kidney disease. Thus, high-titer anti-HBs through vaccination is frequently not achieved. In general, vaccine response rates are 80–95% in healthy individuals [27,28], but only 40–60% in patients undergoing dialysis [29], decreasing with progressive renal dysfunction [30]. These findings support early vaccination, ideally before significant renal impairment, and the consideration of enhanced regimens such as double-dose [23]. Post-transplantation monitoring is equally important. Antibody titers typically decline under immunosuppression, and regular measurements with booster vaccinations are recommended. It should be noted that a decline in anti-HBs titers after initial seroconversion does not necessarily indicate loss of immune memory, and current guidelines do not recommend delaying transplantation solely on this basis [31].

Quantification of HBV-DNA is critical for risk assessment and early diagnosis. The Japanese guidelines, revised in October 2023, recommend DNA monitoring (1, 3, 6, 9, 12, 18, and 24 months) for elective kidney transplantation [23]. While prophylactic nucleoside analog administration is considered for high-risk cases (e.g., those requiring rituximab or intensified immunosuppression), the standard strategy in Japan remains pre-emptive therapy. Antiviral treatment is initiated immediately upon the detection of HBV-DNA [23]. At our institution, management follows this guideline; however, HBV-DNA testing is not reimbursed by national insurance as of May 2026. Future insurance coverage is expected to improve risk stratification and clinical safety.

This study had a few limitations. The assays and analytical platforms used to measure the HBV serological markers changed over the study period. Differences in assay principles, analytical sensitivity, and cutoff definitions across chemiluminescent enzyme immunoassays, chemiluminescent immunoassays, and microparticle enzyme immunoassays may have introduced measurement variability. Therefore, direct comparisons of the absolute antibody values across different periods should be interpreted with caution. Four different assay systems were used to measure anti-HBc and anti-HBs over the 13-year study period, based on different principles (CLEIA, CLIA, and MEIA), and cross-assay comparability could not be assessed. In addition, due to differences in assay principles and platforms, particularly between MEIA and CLIA/CLEIA, cross-validation or conversion between assays is not feasible. However, despite these methodological changes, all assays were clinically validated and routinely used in standardized clinical practice at our institution, and manufacturer-recommended cut-off values were consistently applied to determine positive or negative serological status. Furthermore, antibody titers themselves were not within the scope of this study. Therefore, the impact of inter-assay variability on the primary classification of HBV serological status is likely to be limited. HBV-DNA testing was outsourced until July 2024, when detailed information regarding specific assay platforms and limits of detection was unavailable. Although all tests were based on polymerase chain reaction and interpreted as negative when HBV-DNA was undetectable, subtle differences in analytical sensitivity across assays could not be completely excluded. Transient anti-HBs positivity was observed without corresponding anti-HBc seroconversion in certain cases. Since there were no guidelines for such a population in Japan until 2023, these discrepancies may result from missing data during follow-up or protocol non-adherence in earlier cases. Furthermore, only 15.4% underwent HBV-DNA testing during follow-up for the same reason. Consequently, these findings should be interpreted with caution, although there was no clinically evident transmission. This was a single-center retrospective study with a limited sample size, which may have restricted the generalizability of our findings. Larger multicenter studies are warranted to confirm the safety of kidney transplantation in donors with resolved HBV infection.

## 5. Conclusions

In conclusion, no clinically apparent HBV transmission occurred in our cohort; however, the recipients failed to maintain protective anti-HBs levels. These findings underscore the need for optimized vaccination strategies and systematic post-transplant monitoring to ensure the safety of kidney transplantation in donors with resolved HBV infections.

## Figures and Tables

**Table 1:** Baseline characteristics of donors and recipients.

Donor	
Age, years, median (range)	67.0 (44–77)
Sex (male/female)	11/15
**Recipient**	
Age, years, median (range)	47.5 (23–70)
Sex (male/female)	20/6
Primary renal disease, n (%)	
Diabetes mellitus	9 (34.6)
Glomerulonephritis	6 (23.1)
IgA nephropathy	5 (19.2)
Others	6 (23.1)
Mode of dialysis before KT, n (%)	
HD	10 (38.5)
PD	2 (7.7)
HD + PD	4 (15.4)
PEKT	10 (38.5)
Duration of Dialysis, months, median (range)	26 (1–141)
ABO incompatible, n (%)	12 (46.2)

HD—hemodialysis; IgA—immunoglobulin A; KT—kidney transplantation; PD—peritoneal dialysis; PEKT—preemptive kidney transplantation.

**Table 2:** HBV vaccination history and pre-transplant anti-HBs assessment.

Patient No.	Age	Sex (M/F)	Number of Vaccine Doses	Seroconversion (+/−)	Interval † (Months)
1	58	M	3	−	1.6
2	23	M	3	+	0.8
3	47	M	2	+	NA
4	49	F	2	−	1.4
5	34	M	2	+	NA
6	48	M	3	+	2.5
7	52	M	2	−	1.9
8	47	M	2	NA	1.4
9	47	M	2	−	1.9
10	57	M	2	+	NA
11	64	F	2	NA	2.1
12	47	M	2	−	1.1
13	66	F	2	−	2.1
14	47	M	2	NA	2.4
15	47	M	2	NA	1.2
16	32	M	1	−	2.6
17	46	F	2	−	1.2
18	51	F	2	−	3.0
19	69	F	2	−	1.4
20	49	M	2	−	4.4
21	29	M	2	−	3.0
22	45	M	2	−	5.8
23	25	M	2	−	1.2
24	52	M	2	NA	1.2
25	70	M	2	−	1.9
26	48	M	2	NA	0.7

† Interval from the last vaccination to transplantation anti-HBs (hepatitis B surface antibody); NA—no available data.

**Table 3:** HBV status and graft/patient outcomes after living donor kidney transplantation.

HBV Status and Graft/Patient Outcomes	
Follow-up duration, years, median (range)	6.7 (1.6–13.1)
Seroconversion, n (%)	
HBsAg	0 (0.0)
anti-HBs	5 (19.2)
anti-HBc	0 (0.0)
HBV-DNA	0 (0.0)
Elevated liver enzymes, n (%)	5 (19.2)
Graft loss, n (%)	1 (3.8)
Patient death, n (%)	0 (0.0)

anti-HBc—hepatitis B core antibody; anti-HBs—hepatitis B surface antibody; HBsAg—hepatitis B surface antigen; HBV-DNA—hepatitis B virus deoxyribonucleic acid.

**Table 4:** Summary of published articles on outcomes after kidney transplantation from anti-HBc-positive donors.

Author (Published Year)	Baseline HBV Status of Recipients	Sero- Conversion	Elevated Transaminases	Hepatic Failure	Graft Loss	Death
Satterthwaite R et al. (1997) [7]	Group 1: 27 anti-HBc (−)	18.5%	3.7%	-	24%	12%
	Group 2: 11 anti-HBc (+)	0%	18.2%	-	18%	19%
Madayag RM et al. (1997) [8]	45 vaccinated	26.6%	17.8%	-	11.1%	-
Krieger NR et al. (2001) [9]	Group 1: 7 anti-HBs (+)	0%	17.9%	-	0%	0%
	Group 2: 19 anti-HBc (+)	5.3%		-	20%	0%
	Group 3: 2 HBsAg (+)	-		-	0%	0%
Fong TL et al. (2002) [10]	763 ant-HBc (−)	2.2%	-	-	-	-
Miédougé et al. (2003) [11]	9 anti-HBs (+)	0%	-	-	-	-
De Feo TM et al. (2005) [12]	12 HBsAg (+), 62 naïve, 177 recovered/immunized	0%	-	-	-	-
Veroux M et al. (2005) [13]	Group 1: 28 recovered/immunized	14.2%	17.8%	-	7%	7%
	Group 2: 8 naïve	12.5%	25%	-	0%	0%
De Feo TM et al. (2006) [14]	Group 1: 62 naïve	0%	0%	0%	-	-
	Group 2: 140 vaccinated	2.9%	0%	0%	-	-
	Group 3: 37 recovered	0%	0%	0%	-	-
Mahboobi N et al. (2012) [15]	1385 various	2.3%	-	-	-	-
Chancharoenthana W et al. (2014) [16]	43 anti-HBs (+)	0%	0%	0%	-	-
Abrão JM et al. (2014) [17]	50 anti-HBs (+)	0%	0%	-	-	-
Jeon JM et al. (2018) [18]	Group 1: 356 anti-HBc (−)/anti-HBs (−)	1.1%	-	0%	16.3%	2.8%
	Group 2: 652 anti-HBc (−)/anti-HBs (+)	1.4%	-	0%	17.3%	2.8%
	Group 3: 142 anti-HBc (+)/anti-HBs (−)	5.6%	-	0.7%	16.2%	7.0%
	Group 4: 809 anti-HBc (+)/anti-HBs (+)	1.2%	-	0.1%	17.1%	5.2%
Wang XD et al. (2021) [19]	83 anti-HBc (−)/HBsAg (−)	2.4%	2.4%	-	4.8%	1.2%
Wang XD et al. (2021) [20]	384 anti-HBc (−)/153 anti-HBs (+)	2.6%	11.2%	-	4.9%	1.0%
Yamada R et al. (2022) [21]	45 Naïve	4.4%	8.9%	0%	6.7%	2.2%

anti-HBc—hepatitis B core antibody; anti-HBs—hepatitis B surface antibody; HBsAg—hepatitis B surface antigen; HBV—hepatitis B virus.

## Data Availability

Data are available upon request from the corresponding author, S.Y.

## Abbreviations

The following abbreviations are used in this manuscript:

ALT	alanine aminotransferase
anti-HBc	anti-hepatitis B core antibody
anti-HBs	anti-hepatitis B surface antibody
cccDNA	covalently closed circular deoxyribonucleic acid
HBsAg	hepatitis B surface antigen
HBV	hepatitis B virus
HBV-DNA	hepatitis B virus deoxyribonucleic acid
HD	Hemodialysis
JST	Japan Society for Transplantation
KDIGO	Kidney Disease: Improving Global Outcomes
MMF	mycophenolate mofetil
PCR	polymerase chain reaction
PD	peritoneal dialysis
PEKT	preemptive kidney transplantation
